# Nondestructive Imaging of Manufacturing Defects in Microarchitected Materials

**DOI:** 10.1021/acsaenm.4c00160

**Published:** 2024-04-15

**Authors:** Brian W. Blankenship, Timon Meier, Sophia Lafia Arvin, Jingang Li, Nathan Seymour, Natalia De La Torre, Brian Hsu, Naichen Zhao, Stefanos Mavrikos, Runxuan Li, Costas P. Grigoropoulos

**Affiliations:** † Laser Thermal Laboratory, Department of Mechanical Engineering, University of California, Berkeley, Berkeley, California 94720, United States

**Keywords:** mechanical metamaterials, defects, two-photon polymerization, confocal imaging, polymers

## Abstract

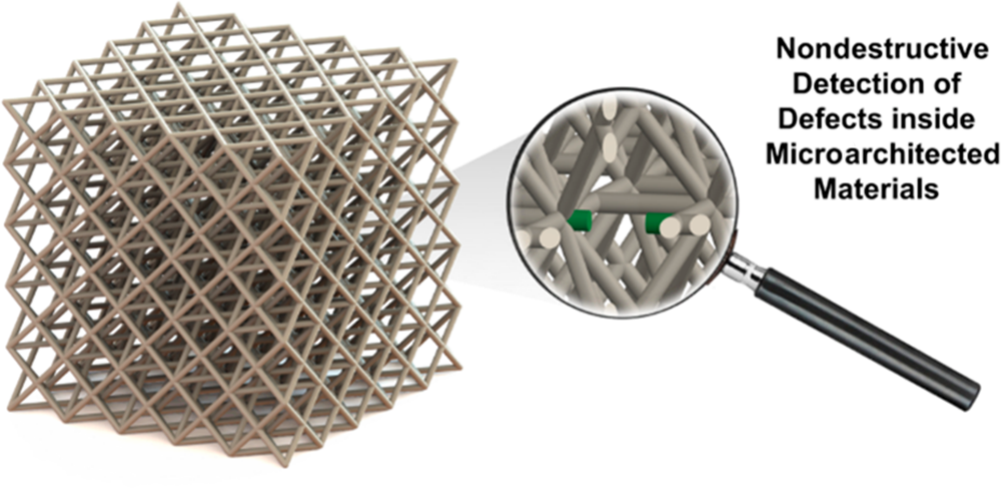

Defects in microarchitected materials exhibit a dual nature, capable of both unlocking innovative functionalities and degrading their performance. Specifically, while intentional defects are strategically introduced to customize and enhance mechanical responses, inadvertent defects stemming from manufacturing errors can disrupt the symmetries and intricate interactions within these materials. In this study, we demonstrate a nondestructive optical imaging technique that can precisely locate defects inside microscale metamaterials, as well as provide detailed insights on the specific type of defect.

## Introduction

3D microarchitected materials represent a transformative class of engineered materials with extraordinary, tailored properties unobtainable in their bulk counterparts.^1,2^ These metamaterials are defined by their intricate microscale and nanoscale geometries.^3,4^ The introduction of periodic voids and lattices can imbue distinct properties including ultrahigh stiffness,^1,5^ exceptional energy absorption,^2,6^ unconventional acoustic dispersion,^7,8^ and auxeticity.^9^ The utility and versatility of these materials has propelled their adoption into commercial applications such as biomedical stents, protective helmets, frequency-selective soundproofing materials, and footwear.^10,11^

The rapidly evolving landscape of metamaterial design is increasingly underpinned by the advances in machine learning and geometry generation tools—a trend that is reshaping the exploration of material geometries with unprecedented ease and efficiency.^9,12^ This paradigm shift is notably driven by the enhanced accessibility and sophistication of machine learning algorithms, which become integral in the iterative process of designing, simulating, and optimizing the complex and unintuitive morphologies inherent to metamaterials.

Concurrently, the development of mesoscale additive manufacturing techniques enables the fabrication of millimeter-scaled parts with microscale and nanoscale features.^13,14^ Rapidly growing demand for higher throughput manufacturing techniques is often accompanied by an increased risk of printing defects and manufacturing errors.^14^ These anomalies include voids where material failed to properly polymerize, improperly stitched interfaces resulting in offset unit cells, and even unintended formation of extra beam members within a unit cell. Although intentional defects can sometimes be interesting and beneficial to the functionality and properties of a material, they can be problematic when their specific, uncontrolled incorporation significantly alters their functional properties.^2,15^

However, detecting defects in microarchitected materials is challenging due to their subtle presence within complex, often large periodic structures of the printed material. These materials have been both indirectly probed with laser vibrometry and mechanical testing and directly imaged by scanning electron microscopy (SEM) and X-ray tomography.^16−19^ With laser vibrometry, while nondestructive, it is difficult to pinpoint the nature or exact location of defects across a lattice.^17^ Mechanical characterization can provide some insights into the presence of defects but often compromises the integrity of the structure upon examination.

Additionally, traditional imaging techniques, including SEM and X-ray tomography, offer a more direct examination of these structures.^16,20^ However, they also come with their own drawbacks. X-ray tomography provides a comprehensive view of internal structures but can be limited by achievable resolution and access to radiation sources.^21^ While SEM is capable of adequately resolving nanoscale features with high resolution, it is limited to surface analysis and cannot effectively penetrate the interior sections of the material. These challenges prompt the development of rapid, nondestructive, and high-resolution imaging techniques to accurately detect defects in metamaterials.

Confocal microscopy, a widely used tool in biological imaging, effectively addresses each of these challenges by providing nondestructive imaging of the entire structure at submicrometer resolutions. Our previous study has shown both the ability to render microarchitected materials with reasonably accurate dimensions and observe their external and internal deformation during *in situ* mechanical loading.^22^ In this work, we enhance the versatility of this technique by demonstrating its capacity to precisely identify both the location and the nature of incorporated manufacturing defects within the lattice.

## Results and Discussion

**Figure 1:**
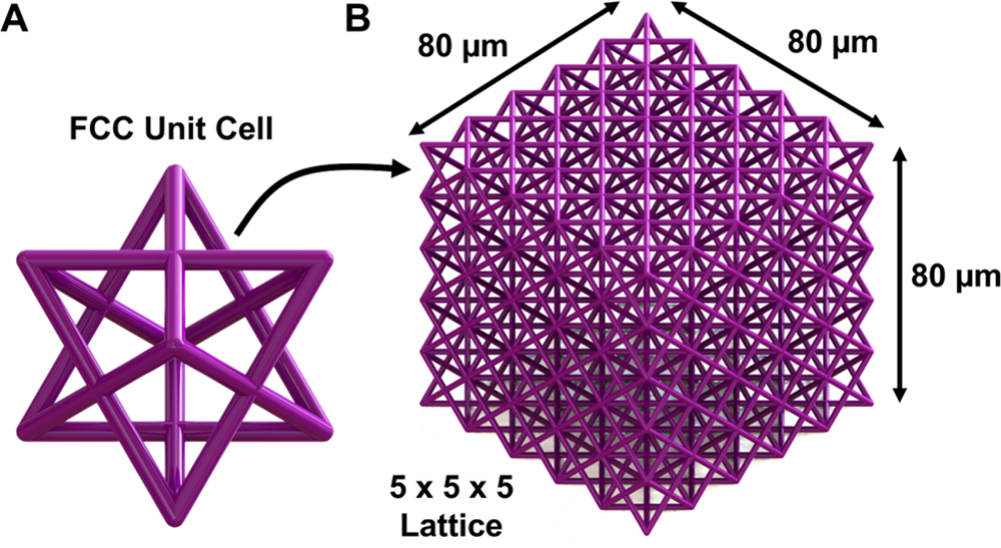
Schematic of the lattice structure. (A) 16 μm × 16 μm × 16 μm cell modeled after a FCC crystal cell. (B) Patterned into a 5 × 5 × 5 lattice where a complex arrangement of beam members shields internal members from view.

**Figure 2:**
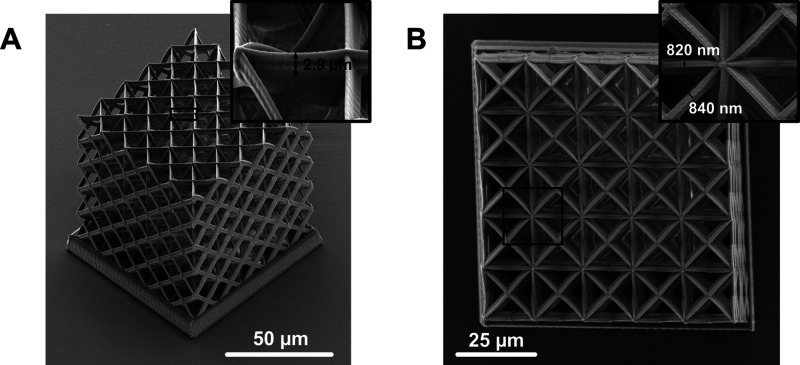
SEM images of the printed metamaterials. (A) Orthogonal view and (B) top view of the microarchitected material fabricated by two-photon polymerization. The insets show the close-up images of the beam members with axial dimensions of roughly 2.9 μm and 820–840 nm. In both images, external beam members conceal internal members.

We first fabricate pristine structures without intentional defects resembling the lattice design displayed in Figure 1 by using a custom-built two-photon polymerization (TPP) printer. This lattice structure is populated by unit cells that resemble an FCC crystal, where thin beams connect the nodes of the unit cell. These structures are fabricated from the standard photosensitive resin, SZ2080.^23^ These structures are printed onto a solid platform to ensure adhesion to the substrate and provide a consistent reference plane for imaging.^24^ SEM images of the printed structures from the orthogonal and top views are shown in Figure 2. The insets of Figure 2A and B depict measurements of the axial and lateral dimension of the prints, which were measured to be on the order 3.0 ± 0.2 μm and 860 ± 60 nm, respectively. Overall, the fabricated samples closely align with the target geometry, with the notable exception that the axial beam dimensions are elongated, which is an inherent consequence of the TPP process.^23,25^

Confocal microscopy is an optically sectioned imaging technique that relies on collecting fluorescence from localized excitation of fluorophores.^26^ To eliminate out-of-focus fluorescence, a pinhole aperture at the detector is employed. This approach enables the capture of high-resolution images from successive depths, facilitating the computational reconstruction of a comprehensive three-dimensional representation of the sample, including their internal features. In real imaging systems, considering optical aberrations and scattering, the achievable lateral and axial resolutions can reach the order of 200–600 and 600–1000 nm, respectively, with high numerical aperture objectives.^27^

In this experimental setup, the formation of the image relies on spatially detecting fluorescence emitted from the microarchitected material at successive depths. This requires that the base material is both transparent and either autofluorescent or suitably functionalized with fluorescent particles or dyes.^22^ These requirements immediately suggest that many metallic or ceramic materials may not be conducive to this imaging process due to their opacity. Nevertheless, a broad spectrum of photopolymerizable materials is known to fulfill these requirements, making them suitable candidates for this technique.

**Figure 3:**
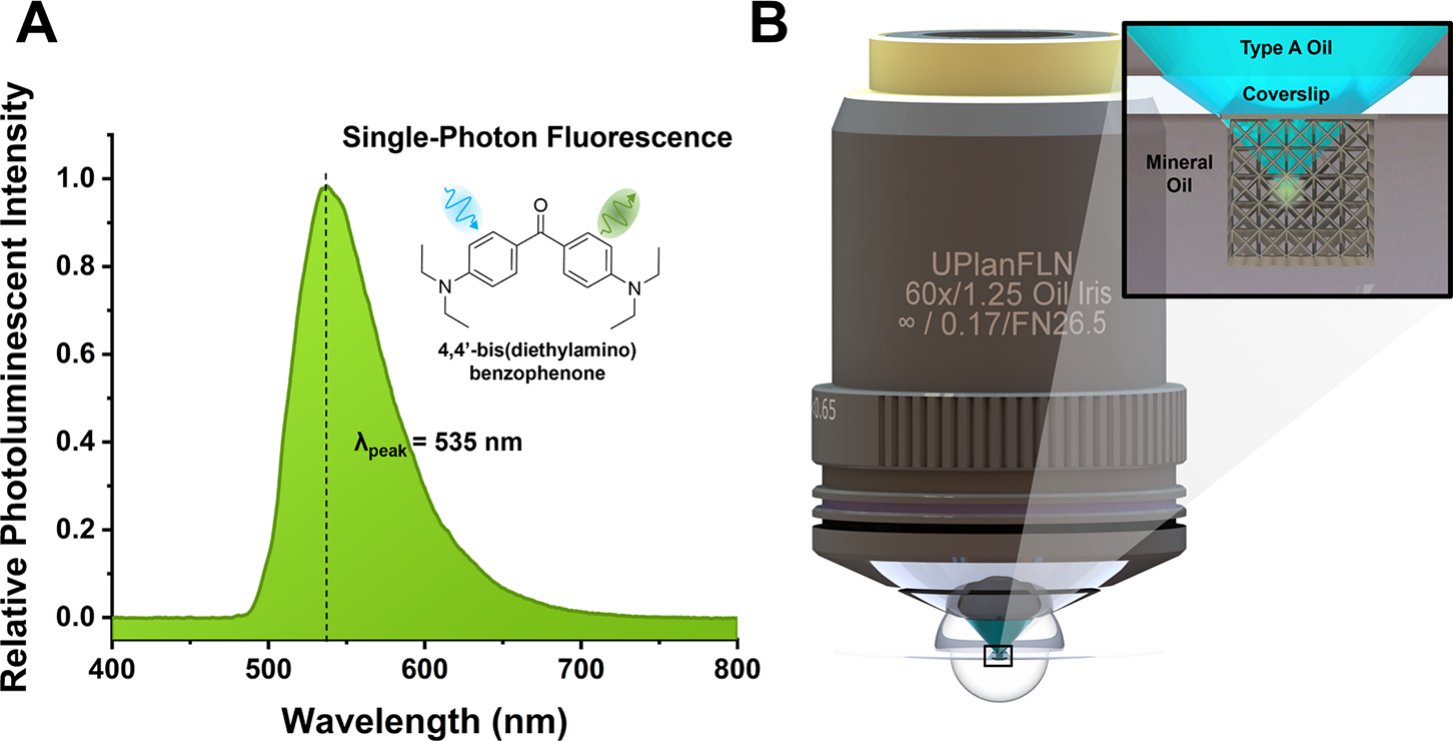
Fluorescence imaging. (A) The measured single-photon fluorescence spectrum of polymerized SZ2080 structures. The bulk of the fluorescence is emitted from the photoinitiator 4,4′-bis(diethylamino)benzophenone (shown in the inset). (B) The optical setup for imaging was with an oil immersion objective lens. The polymerized structure is enveloped in mineral oil that roughly matches the refractive index of the polymer to mitigate scattering.

We measure the photoluminescent spectrum of polymerized SZ2080 material that were processed under the conditions described in the Materials and Method Section. This spectrum, shown in Figure 3A, depicts a broad peak with a peak wavelength at 535 nm. The majority of the collected fluorescence originates from residual 4,4′-bis(diethylamino)benzophenone that remains in the polymer after fabrication. It is also known that the choice and concentration of solvent and resin components affect the fluorescent spectra of this material.^28^ The full effect of the laser processing parameters on the fluorescence spectra is less understood but may still be a deciding factor.

Continuous-wave excitation at 488 nm can produce images with high signal-to-background contrast at low <100 μW power.^22^ In these investigations, we use a 60X 1.25 NA oil immersion lens to image the structures. The complex structure of unit cells and beam members in microarchitected materials results in intricate spatial variations in refractive index from the polymer (NA ≈ 1.48) and surrounding air (NA ≈ 1). Thus, scattering quickly dominates after imaging into the structure. In order to mitigate this scattering, the structures are enveloped in a layer of oil that roughly matches the refractive index of the printed material. A diagram of this imaging setup is shown in Figure 3B. In turn, this enhances the resolvable imaging depth into the lattice. Additional plots of the collected fluorescence intensity as a function of depth are shown in Figure S1.

Figure S2 depicts rendering of a pristine lattice alongside sectioned views revealing the interior and horizontal z-slices taken by the confocal microscope. Lateral fwhm of the imaged beam elements in the structure were measured to be 1.18 ±. 08 μm (see Figure S3), which are expectedly larger than the features measured by the SEM. However, it has been shown that these images can be computationally improved using filters, thresholding, erosion, and elongation to create renderings that are reasonably accurate relative to a datum such as in our case the SEM images.

**Figure 4:**
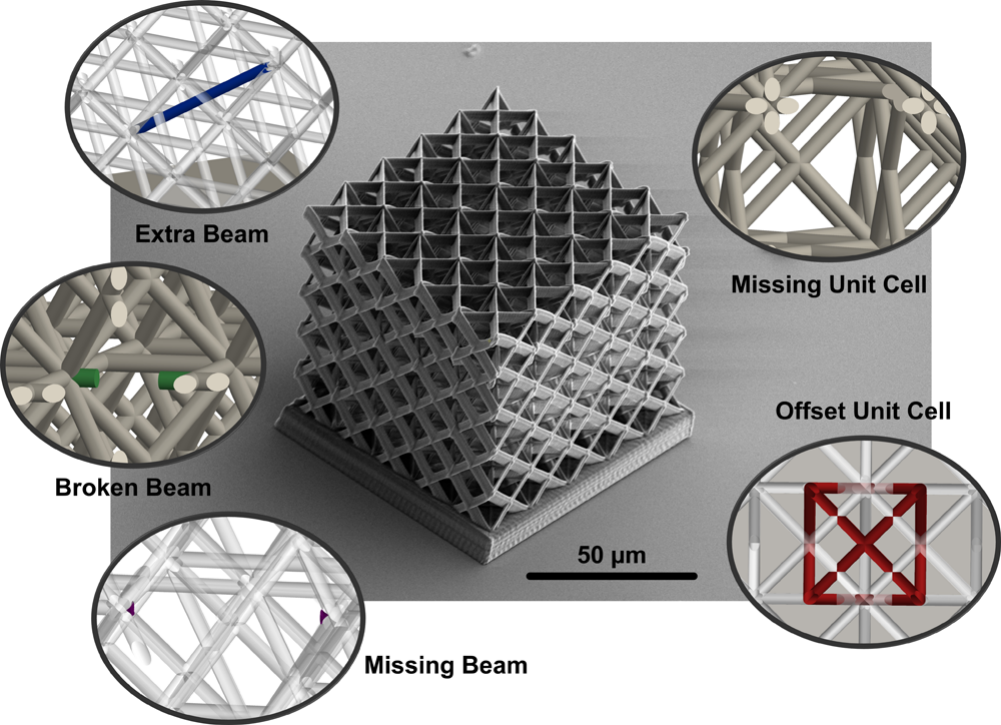
Defects in microarchitected materials. SEM image of a lattice with five types of common defects is shown in the center. By inspection, it is nearly impossible to locate the location of individual defects or the type. On the sides, renderings of individual defects within the structure are highlighted.

We intentionally introduce five common types of defects into the interior of the lattice structure that we subsequently print using the methodology that is outlined previously. These defects include (1) missing beam members from unit cells, (2) voided cells, (3) incorporation of extra beam members in a unit cell, (4) offset unit cells from poor stitching, and (5) incomplete beams. Visualizations of each of these defects, as they are present in the lattice, are shown in Figure 4. As depicted in the SEM image in the center, these defects are largely unnoticeable.

**Figure 5:**
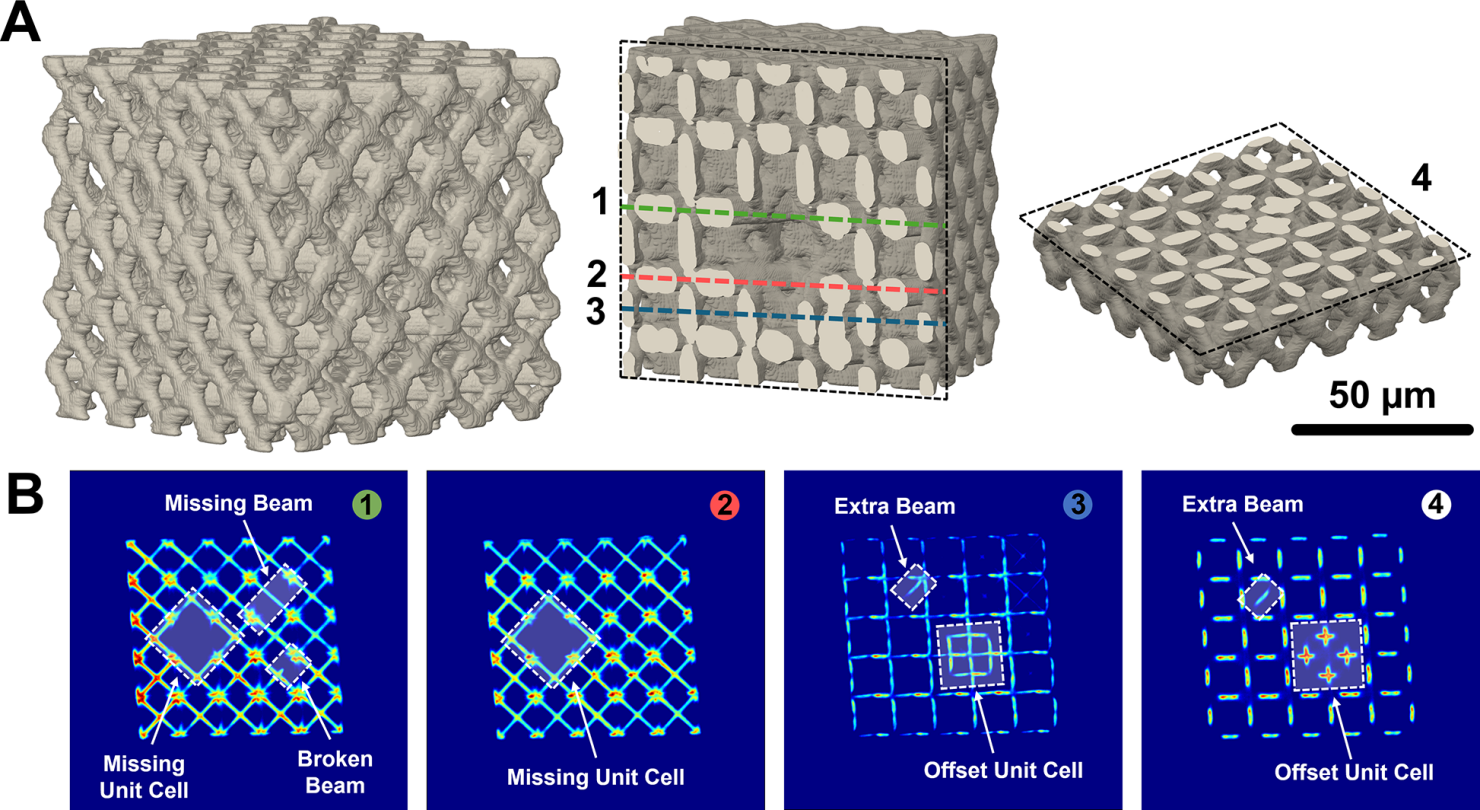
Defect imaging. (A) Rendering of a lattice with incorporated defects as imaged by a confocal microscope. Selected cross sectional views of the rendering show missing defects (middle) as well as offset unit cells and extra beam members (right). (B) Confocal microscopy slices effectively capture each of the defects present in the lattice.

Furthermore, the fabricated “defect” structures are imaged under conditions identical with those of the pristine structures. Renderings of these structures are presented in Figure 5, which includes z-slices that effectively highlight each of the defects. The confocal images clearly reveal defects lying parallel to the z-slices, while out-of-plane anomalies, such as extra beams, are made more evident in the sectional views of the reconstructions. Remarkably, optical imaging through confocal microscopy unveils the internal details of these structures with clarity unattainable by SEM, and it does so without the necessity for high-energy synchrotron radiation typically required for such detailed resolution. This demonstrates the potential of confocal microscopy as a diagnostic tool for detecting defects in microarchitected materials.

After imaging, the structures are immersed in chloroform to remove the mineral oil. Widefield images before and after dissolution in chloroform are shown in Figure S5. Whether or not inclusion of mineral oil or immersion in chloroform influences the mechanical or optical properties of the polymerized material has yet to be determined.

As we seek to extend this technique to larger structures, it is important to understand the limits of imaging depth, particularly considering the trade-offs between depth and resolution. Generally, lenses with higher numerical apertures, capable of resolving smaller features, are constrained by shorter working distances, thus limiting the maximum observable height. Most commercial oil objectives have working distances in the range of 150–200 μm. Additionally, imaging depth can be restricted by scattering, with signal intensity exhibiting a nonlinear decrease as a function of depth for a constant excitation power. This implies that for larger structures, adjusting the laser power according to depth could be beneficial to maintain a high signal-to-background ratio. However, from data shown in Figure S1, the contrast between signal and background increases with imaging depth despite efforts to minimize scattering through refractive index matching. This observation suggests that scattering can also be a limiting factor.

Similarly framed problems with scattering in biological imaging have been addressed by using multiphoton imaging, which limits absorption of light through the media and experiences less scatter by use of longer excitation wavelengths. However, the resolution of single-photon fluorescence imaging is generally better than multiphoton confocal imaging by a factor of ≈√2 given its use of longer excitation wavelengths which may factor in its overall effectiveness of investigating sufficiently small features in microarchitected materials.

While we image these microarchitected materials after they have been developed, there is a compelling opportunity to apply this principle to image structures during the fabrication process. It is expected that other techniques such as coherent anti-Stokes Raman spectroscopy could be integrated to distinguish local polymerization characteristics that would provide real time feedback on the efficacy of processing parameters.^29,30^ Ultimately, this will introduce new complexities to imaging processing and analysis.

Defects play a critical role in the functional integrity of microarchitected materials, making it imperative to detect and manage unwanted anomalies. Our study presents a robust technique for nondestructively identifying defects inside of a prototypical microarchitected material using optical imaging techniques. In our demonstration, we incorporate five different types of intentional defects into an engineered lattice structure and use confocal microscopy to directly determine the location and nature of each defect. This method not only enhances the existing array of diagnostic techniques but also opens the door for adapting and applying similar strategies during the fabrication process itself, enabling the *in situ* detection and characterization of defects.

## Materials and Methods

We utilize a recipe for preparing the hybrid organic–inorganic resin, SZ2080, taken and modified from Ovsianikov et al.^23^ The resin is composed of 70 wt % zirconium n-propoxide and 10 wt % (2-dimethylaminoethyl) methacrylate. The structures are approximately 1% v/v 4,4′-bis(diethylamino)benzophenone.

Structures were fabricated using two-photon polymerization on an SZ2080 photoresist. Laser light from a FemtoFiber Pro NIR laser, which emits 780 nm, 100 fs fwhm, pulses at 80 MHz is focused through a (100×/1.3 NA) oil objective lens (Zeiss). The laser output energy was measured before the objective lens at 4.2 mW. The structures are written by positioning a three-axis piezo stage relative to the focus of the laser beam.

Coverslips of printed material are immersed in 4-methyl-2-pentanone for 30 min to dissolve the nonpolymerized resist and 1-propanol for 10 min for rinsing.

We utilized a Bruker Swept-Field Confocal microscope for imaging using an excitation wavelength of 488 nm and using a 488 nm long pass filter. We employed a 35 μm slit aperture. The camera images a 512 × 512 array of pixels with 16-bit intensity resolution. Images were taken in 200 nm slices (roughly 400 images). Excitation power was limited to below 100 μW.

PL measurements were taken with an Ocean Optics USB4000 spectrometer.

## Supporting Information Available

Signal intensity vs imaging depth plots, confocal slices of pristine structures, line width measurements, renderings of the “defect” structure, widefield images before and after removal of refractive index matching fluid (PDF)

## References

[ref1] Zheng X.; Lee H.; Weisgraber T. H.; Shusteff M.; DeOtte J.; Duoss E. B.; Kuntz J. D.; Biener M. M.; Ge Q.; Jackson J. A.; Kucheyev S. O.; Fang N. X.; Spadaccini C. M. Ultralight, Ultrastiff Mechanical Metamaterials. Science 2014, 344 (6190), 1373–1377. 10.1126/science.1252291. 24948733

[ref2] Vangelatos Z.; Sheikh H. M.; Marcus P. S.; Grigoropoulos C. P.; Lopez V. Z.; Flamourakis G.; Farsari M. Strength through Defects: A Novel Bayesian Approach for the Optimization of Architected Materials. Science Advances 2021, 7 (41), eabk2218 10.1126/sciadv.abk2218. 34623909 PMC8500519

[ref3] Jiao P.; Mueller J.; Raney J. R.; Zheng X.; Alavi A. H. Mechanical Metamaterials and Beyond. Nat. Commun. 2023, 14 (1), 6004 10.1038/s41467-023-41679-8. 37752150 PMC10522661

[ref4] Bauer J.; Meza L. R.; Schaedler T. A.; Schwaiger R.; Zheng X.; Valdevit L. Nanolattices: An Emerging Class of Mechanical Metamaterials. Adv. Mater. 2017, 29 (40), 1701850 10.1002/adma.201701850. 28873250

[ref5] do Rosário J. J.; Lilleodden E. T.; Waleczek M.; Kubrin R.; Petrov A. Yu.; Dyachenko P. N.; Sabisch J. E. C.; Nielsch K.; Huber N.; Eich M.; Schneider G. A. Self-Assembled Ultra High Strength, Ultra Stiff Mechanical Metamaterials Based on Inverse Opals. Adv. Eng. Mater. 2015, 17 (10), 1420–1424. 10.1002/adem.201500118.

[ref6] Portela C. M.; Edwards B. W.; Veysset D.; Sun Y.; Nelson K. A.; Kochmann D. M.; Greer J. R. Supersonic Impact Resilience of Nanoarchitected Carbon. Nat. Mater. 2021, 20 (11), 1491–1497. 10.1038/s41563-021-01033-z. 34168332

[ref7] Guo Y.; Rosa M. I. N.; Gupta M.; Dolan B. E.; Fields B.; Valdevit L.; Ruzzene M. Minimal Surface-Based Materials for Topological Elastic Wave Guiding. Adv. Funct. Mater. 2022, 32 (30), 2204122 10.1002/adfm.202204122.

[ref8] Bayat A.; Gaitanaros S. Wave Directionality in Three-Dimensional Periodic Lattices. Journal of Applied Mechanics 2018, 85 (1), 011004 10.1115/1.4038287.

[ref9] Meier T.; Li R.; Mavrikos S.; Blankenship B.; Vangelatos Z.; Yildizdag M. E.; Grigoropoulos C. P. Obtaining Auxetic and Isotropic Metamaterials in Counterintuitive Design Spaces: An Automated Optimization Approach and Experimental Characterization. npj Comput. Mater. 2024, 10 (1), 1–12. 10.1038/s41524-023-01186-2.

[ref10] Yang M.; Sheng P. Acoustic Metamaterial Absorbers: The Path to Commercialization. Appl. Phys. Lett. 2023, 122 (26), 260504 10.1063/5.0147941.

[ref11] Xue H.; Luo Z.; Brown T.; Beier S. Design of Self-Expanding Auxetic Stents Using Topology Optimization. Frontiers in Bioengineering and Biotechnology 2020, 8, na 10.3389/fbioe.2020.00736. PMC7381139 32766219

[ref12] Ha C. S.; Yao D.; Xu Z.; Liu C.; Liu H.; Elkins D.; Kile M.; Deshpande V.; Kong Z.; Bauchy M.; Zheng X. Rapid Inverse Design of Metamaterials Based on Prescribed Mechanical Behavior through Machine Learning. Nat. Commun. 2023, 14 (1), 5765 10.1038/s41467-023-40854-1. 37718343 PMC10505607

[ref13] Jonušauskas L.; Gailevičius D.; Rekštytė S.; Baldacchini T.; Juodkazis S.; Malinauskas M. Mesoscale Laser 3D Printing. Opt. Express, OE 2019, 27 (11), 15205–15221. 10.1364/OE.27.015205. 31163720

[ref14] Somers P.; Liang Z.; Johnson J. E.; Boudouris B. W.; Pan L.; Xu X. Rapid, Continuous Projection Multi-Photon 3D Printing Enabled by Spatiotemporal Focusing of Femtosecond Pulses. Light: Science & Applications 2021, 10 (1), 199 10.1038/s41377-021-00645-z. PMC8463698 34561417

[ref15] Meeussen A. S.; Oguz E. C.; Shokef Y.; Hecke M. v. Topological Defects Produce Exotic Mechanics in Complex Metamaterials. Nat. Phys. 2020, 16 (3), 307–311. 10.1038/s41567-019-0763-6.

[ref16] Tancogne-Dejean T.; Spierings A. B.; Mohr D. Additively-Manufactured Metallic Micro-Lattice Materials for High Specific Energy Absorption under Static and Dynamic Loading. Acta Mater. 2016, 116, 14–28. 10.1016/j.actamat.2016.05.054.

[ref17] Kai Y.; Dhulipala S.; Sun R.; Lem J.; DeLima W.; Pezeril T.; Portela C. M. Dynamic Diagnosis of Metamaterials through Laser-Induced Vibrational Signatures. Nature 2023, 623 (7987), 514–521. 10.1038/s41586-023-06652-x. 37968526

[ref18] Vangelatos Z.; Komvopoulos K.; Grigoropoulos C.P. Vacancies for Controlling the Behavior of Microstructured Three-Dimensional Mechanical Metamaterials. Mathematics and Mechanics of Solids 2019, 24 (2), 511–524. 10.1177/1081286518810739.

[ref19] Meza L. R.; Phlipot G. P.; Portela C. M.; Maggi A.; Montemayor L. C.; Comella A.; Kochmann D. M.; Greer J. R. Reexamining the Mechanical Property Space of Three-Dimensional Lattice Architectures. Acta Mater. 2017, 140, 424–432. 10.1016/j.actamat.2017.08.052.

[ref20] Sheikh H. M.; Meier T.; Blankenship B.; Vangelatos Z.; Zhao N.; Marcus P. S.; Grigoropoulos C. P. Systematic Design of Cauchy Symmetric Structures through Bayesian Optimization. International Journal of Mechanical Sciences 2022, 236, 107741 10.1016/j.ijmecsci.2022.107741.

[ref21] Hu W.; Cao X.; Zhang X.; Huang Z.; Chen Z.; Wu W.; Xi L.; Li Y.; Fang D. Deformation Mechanisms and Mechanical Performances of Architected Mechanical Metamaterials with Gyroid Topologies: Synchrotron X-Ray Radiation in-Situ Compression Experiments and 3D Image Based Finite Element Analysis. Extreme Mechanics Letters 2021, 44, 101229 10.1016/j.eml.2021.101229.

[ref22] Blankenship B. W.; Meier T.; Zhao N.; Mavrikos S.; Arvin S.; De La Torre N.; Hsu B.; Seymour N.; Grigoropoulos C. P. Three-Dimensional Optical Imaging of Internal Deformations in Polymeric Microscale Mechanical Metamaterials. Nano Lett. 2024, 24, 2735 10.1021/acs.nanolett.3c04421. 38277644 PMC10921468

[ref23] Ovsianikov A.; Viertl J.; Chichkov B.; Oubaha M.; MacCraith B.; Sakellari I.; Giakoumaki A.; Gray D.; Vamvakaki M.; Farsari M.; Fotakis C. Ultra-Low Shrinkage Hybrid Photosensitive Material for Two-Photon Polymerization Microfabrication. ACS Nano 2008, 2 (11), 2257–2262. 10.1021/nn800451w. 19206391

[ref24] Blankenship B. W.; Jones Z.; Zhao N.; Singh H.; Sarkar A.; Li R.; Suh E.; Chen A.; Grigoropoulos C. P.; Ajoy A. Complex Three-Dimensional Microscale Structures for Quantum Sensing Applications. Nano Lett. 2023, 23, 9272 10.1021/acs.nanolett.3c02251. 37811908 PMC10603797

[ref25] Zhou X.; Hou Y.; Lin J. A Review on the Processing Accuracy of Two-Photon Polymerization. AIP Advances 2015, 5 (3), 030701 10.1063/1.4916886.

[ref26] Elliott A. D. Confocal Microscopy: Principles and Modern Practices. Curr. Protoc Cytom 2020, 92 (1), e68 10.1002/cpcy.68. 31876974 PMC6961134

[ref27] Fouquet C.; Gilles J.-F.; Heck N.; Dos Santos M.; Schwartzmann R.; Cannaya V.; Morel M.-P.; Davidson R. S.; Trembleau A.; Bolte S. Improving Axial Resolution in Confocal Microscopy with New High Refractive Index Mounting Media. PLoS One 2015, 10 (3), e0121096 10.1371/journal.pone.0121096. 25822785 PMC4379090

[ref28] Shoute L. C. T. Dual Fluorescence of 4,4′-Bis(Dimethylamino)Benzophenone. Effects of Specific and Nonspecific Interaction on the Formation of Twisted Intramolecular Charge Transfer. Chem. Phys. Lett. 1992, 195 (2), 255–261. 10.1016/0009-2614(92)86145-8.

[ref29] LaFratta C. N.; Baldacchini T. Two-Photon Polymerization Metrology: Characterization Methods of Mechanisms and Microstructures. Micromachines (Basel) 2017, 8 (4), 101 10.3390/mi8040101.

[ref30] Baldacchini T.; Zimmerley M.; Kuo C.-H.; Potma E. O.; Zadoyan R. Characterization of Microstructures Fabricated by Two-Photon Polymerization Using Coherent Anti-Stokes Raman Scattering Microscopy. J. Phys. Chem. B 2009, 113 (38), 12663–12668. 10.1021/jp9058998. 19715350 PMC3972915

