# Supplementary material for: Nondestructive Imaging of Manufacturing Defects in Microarchitected Materials
Source: ACS Appl Eng Mater. 2024 Apr 15;2(7):1737–42. doi: 10.1021/acsaenm.4c00160 (PMC11287491; doi:10.1021/acsaenm.4c00160)
Supplement: Supplementary file 1 — em4c00160_si_001.pdf [file em4c00160_si_001.pdf]

Supporting Information for:

Non-Destructive Imaging of Manufacturing Defects in Micro-Architected Materials

Brian W. Blankenship[1†], Timon Meier[1†], Sophia Lafia Arvin[1], Jingang Li[1], Nathan Seymour[1], Natalia De La Torre[1], Brian Hsu[1], Naichen Zhao[1], Stefanos Mavrikos[1], Runxuan Li[1], Costas P. Grigoropoulos[1*]

1. Laser Thermal Laboratory, Department of Mechanical Engineering, University of California, Berkeley, CA 94720, USA

† These Authors contributed equally

*Corresponding Author

Email: cgrigoro@berkeley.edu

Confocal Imaging

The measured image can be understood as the convolution of the object, O(x, y, z) with the point spread function (PSF) of the confocal microscope PSF(x, y, z):

$$I(X, Y, Z)= \int_{-\infty}^{\infty}\int_{-\infty}^{\infty}\int_{-\infty}^{\infty} O(x, y, z) \times PSF_{conf}(x-x', y-y', z-z')dx'dy'dz'$$

$$\equiv O(x, y, z) \otimes PSF_{conf}(x, y, z) \quad (1)$$

Where X, Y, Z and x, y, z are the spatial coordinates in image and object space respective.

The point spread function of the confocal microscope is the product of the excitation and detection PSF:

$$PSF_{conf}(x,y,z) = PSF_{exc}(x,y,z) \cdot PSF_{det}(x,y,z) \quad (2)$$

According to the Rayleigh criterion, this would suggest that the upper bound of the resolution of the confocal microscope is roughly constrained by:

$$\text{Lateral Resolution} = \frac{1}{\sqrt{1+(\frac{\lambda_{ex}}{\lambda_{em}})^2}} * \frac{.61\,\lambda_{ex}}{NA} \quad (3)$$

$$\text{Axial Resolution} = \frac{1}{\sqrt{1+\left(\frac{\lambda_{ex}}{\lambda_{em}}\right)^2}} * \frac{2\eta\,\lambda_{ex}}{NA^2} \quad (4)$$

Where η is the refractive index of the medium, NA is the numerical aperture of the objective lens, λ_{ex} is the wavelength of the excitation source, and λ_{em} is the wavelength of the emitted fluorescence that is measured by the detector.

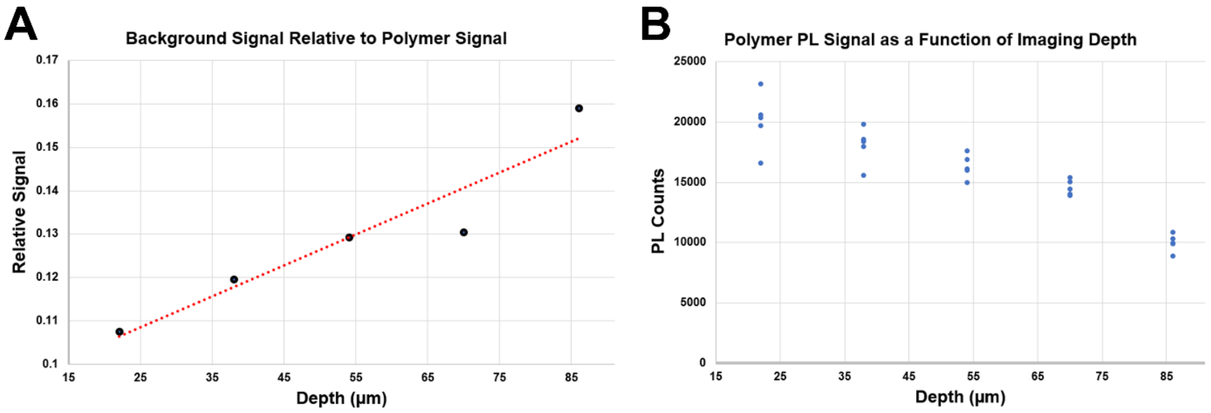

Figure S1: A) Background fluorescence signal relative to the fluorescent polymer signal measured via confocal microscopy as a function of depth. The "background signal" was measured at locations between unit cells on successive layers where there was no polymer present, while "polymer signal" was measured at the same locations on successive layers. Depth is measured assuming there is a 6 μm base on each structure, and unit cells are spaced 16 μm apart. We see that the relative background signal to polymer signal increases with depth which suggests that this technique may be limited by contrast in deeper structures. **B)** Polymer photoluminescence signal as a function of depth for 5 measurements on each layer of similar geometry. These slices are shown in **Figure S2**.

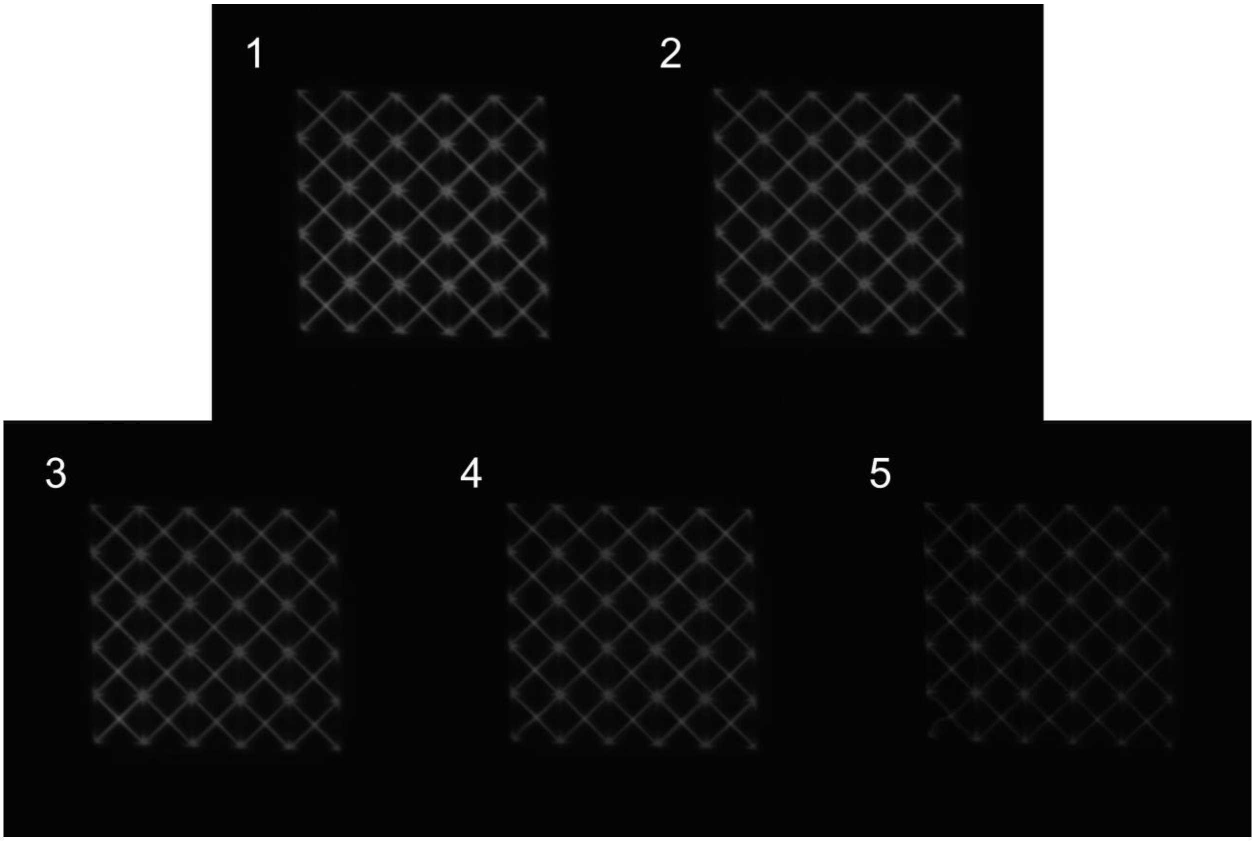

Figure S2: Confocal slices of sections of a unit cell in 5 successive layers of a pristine structure. Beyond a decrease in intensity, the slices appear very similar, and there are negligible changes to the linewidth as a function of depth. Although no intentional defects were placed in the structure, on the bottom left side of layer five there is a small defect from buckling.

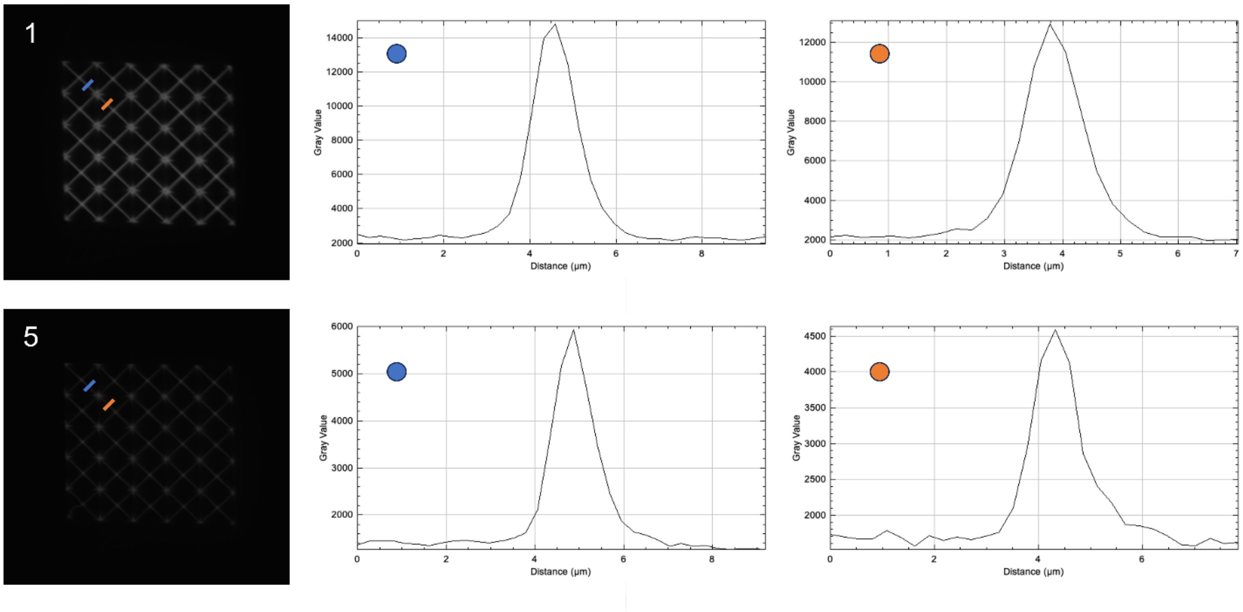

Figure S3: Linewidth measurements taken from the highlighted sections of the confocal slices in layers 1 and 5. The average linewidth (measured from 5 sections) in layer 1 was determined to be 1.21 ± .044 µm whereas the average linewidth in layer 5 was determined to be 1.08 ± .040 µm.

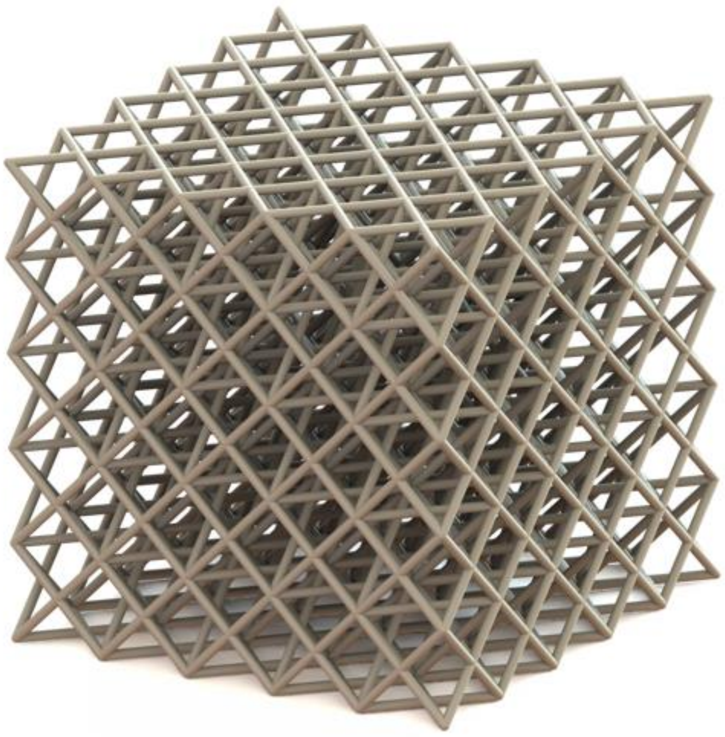

Figure S4: Renderings of the “defect” structure printed and imaged in **Figure 4**. Even with thinner axial beam dimensions, the defects are not visible.

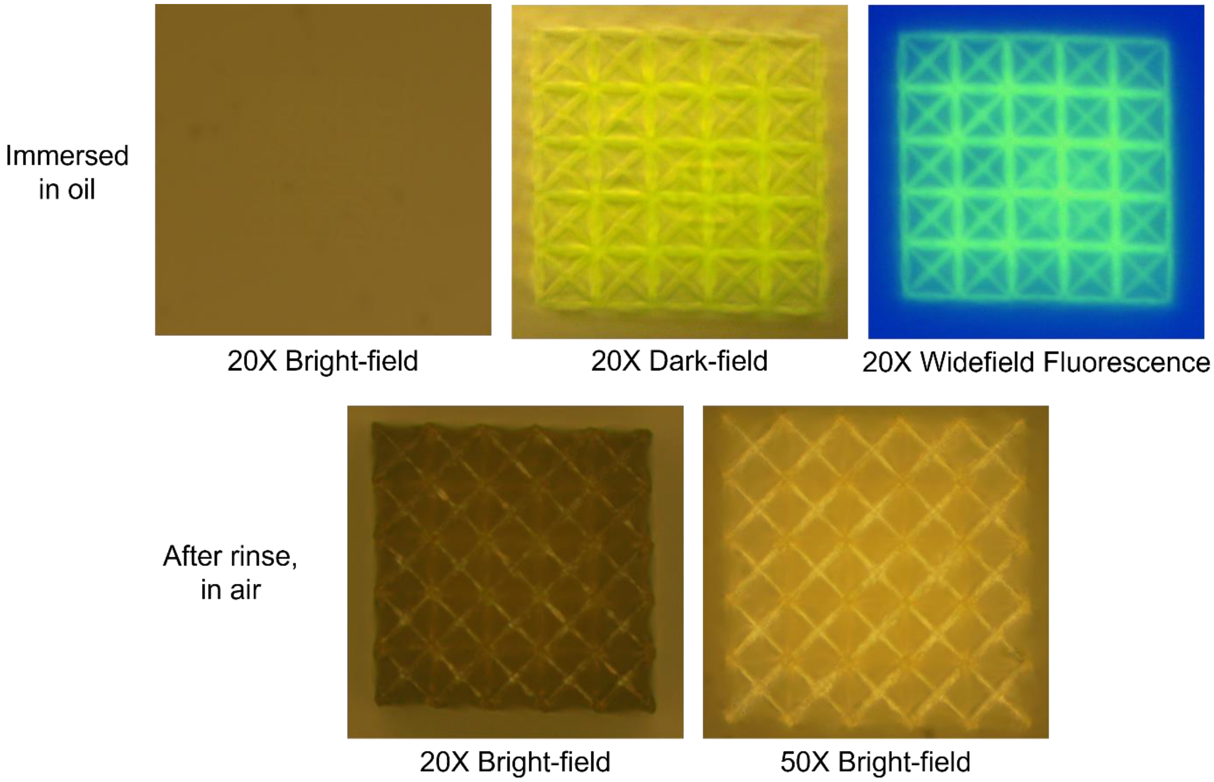

Figure S5: Various images of defect structures immersed in oil (top) and after rinsing with chloroform (bottom). (Top) (left) Widefield images of structures in oil lack contrast in bright-field imaging modalities because of the refractive index matching fluid and transparency of the resin. These structures become visible in dark-field (middle) and fluorescence (right) imaging modalities. (Bottom) After being rinsed with chloroform, the structures appear to retain their original shape with seemingly minimal effect on the geometry of the structures. 20X and 50X denote the magnification of the objective lens used to take the image.
